# A Standard Dictionary of the English Language

**Published:** 1895-05

**Authors:** 


### BOOK NOTICES.

A Standard Dictionary of the English Language upon original plans, designed to give in complete and accurate statement, in the light of the most recent advances in knowledge and in the readiest form for popular use, the orthography, pronunciation, meaning, and etymology of all the words, and the meaning of idiomatic phrases in the speech and literature of the English-speaking peoples. Prepared by more than two hundred specialists and other scholars under the supervision of Isaac K. Funk, D. D., Editor-in-Chief; Francis A. March, LL. D., C. H. D., Consulting Editor; Daniel S. Gregory, D. D., Managing Editor. Associate Editors: John Denison Champlin, M. A.; Rossiter Johnson, Ph. D., LL. D., and Arthur E. Bostwick, Ph. D. Vol. II, M to Z. New York: Funk & Wagnalls Company, London and Toronto, 1895. Printed in the United States. Sold only by subscription. Prices, single-volume edition: half Russia, $12.00; full Russia, with Denison's Patent Reference Index, $14.00; full Morocco, with Denison's Patent Reference Index, $18.00. Two-volume edition: half Russia, $15.00; full Russia, with

Denison's Patent Reference Index, $22.00; full Morocco, with Denison's Patent Reference Index, $22.00. Funk & Wagnalls, 30 Lafayette Place, New York; 44 Fleet Street, London; 11 Richmond Street, W., Toronto.

In THE HOMŒOPATHIC PHYSICIAN for April, 1894, at page 123, was given an elaborate review of the first volume of this great dictionary. In nearly all the subsequent issues of this journal, shorter articles, giving glimpses of the special features of the Standard Dictionary, have been published, so that the admirable qualities of this magnificent sample of fine bookmaking have been kept pretty constantly before the eyes of our readers.

Not a tenth part of these qualities has been told, however, and now comes the second volume to complete this fine monument of the printer's art.

The two volumes together comprise 2,338 pages, 5,000 illustrations made expressly for the work, some of them magnificent colored plates.

As stated in the first review, there are over three hundred thousand words, the exact number, as given by the publishers, being 301,865. There were 247 editors and specialists and 500 readers for quotations. All these people were selected from the front rank of English and American scholars. Each is representative of all that is latest and most approved in his own field of exploration and research; each is an expert in his own school of science, literature, art, handicraft, or trade.

The names of all this distinguished army of literary giants and their portraits are given, which must silence some skeptics in certain quarters, who suggest doubts about the probability of any publisher engaging such an army.

Turning now to the pages of the dictionary, we have our attention attracted by the peculiar way in which

1. Derivitives, compounds, and phrases are frequently run in under the root or principal words; as, educational will be found under education, coffee-bean under coffee, dark horse (an unexpected competitor) under horse, electric gun under electric, etc.

It may be difficult at times to determine which is the principal or more important term in a phrase or a compound word. If the term is not to be found under what seems to be the principal element, turn to the next important element of the compound word or term in the phrase.

2. Frequently it will be seen that variant, obsolescent, and obsolete words are run in under the more generally accepted forms.

3. Many classes of words (mainly technical) formed with the prefixes, or combining forms, acantho-, amphi-, electro-, etc., and not often looked for in a general dictionary, will be found in vocabulary type, with definitions, under the prefix or combining form; as, acanthocephala will be found under its combining form acantho-, etc.

4. Prefixes, as anti-, bi-, in-, intra-, mis-, un-, etc., are defined in their vocabulary places, and words formed with them, the meaning of which being obvious from the component parts, are grouped in table forms under their respective prefixes, without definition; as, bicolor is in the table under the prefix bi-, *twice, doubly, two,* + color; hence, *two-color.* Sometimes such words are grouped under a definitive statement, as under prefix mis-.

5. For convenience, in a number of trades, classified tables of trade terms used are given; as, under carpentry, a table of terms peculiar to carpentry; plumbing, terms peculiar to plumbing, etc. These tables might be called, appropriately, *finding-lists;* as, for example, one may not remember the name cramp, but he knows that it is an implement used in carpentry; by turning to the table under carpentry he will most likely recognize it in the list, and then by turning to it in the vocabulary obtain its definition. For partial list of groups and tables, see page 2108.

6. For explanation of abbreviations used in the text see pages 18, 19, and 2309–2315.

7. For explanation of key to pronunciation see page 20; also, see "The Principles and Explanation of the Scientific Alphabet," pages 2104–2107.

Take for example the prefix Homœo, in which we of the new school of medicine are peculiarly interested. We find this word standing by itself without any attendant syllable.

The first thing we notice is the statement that it is derived from the Greek *homoios.* Then comes the definition: "like, similar, a combining form." Then comes a list of words in which it forms the prefix, together with the meanings of these words. On looking them over we count thirty-two all grouped under this one prefix, all with definitions clearly given and printed in heavy black type so as to quickly catch the eye.

Take another example, Homo, meaning the same. Here we find grouped a total of seventy-four words under this prefix!

Let us turn now to Hydro. This word forms the prefix for one hundred and ninety-three words grouped under it. This is exclusive of a host of principal words arranged in their proper alphabetical place in the ordinary way; such words as hydrocarbon, hydrodynamic, hydrogen, hydrography, etc.

There are scores of thousands of *new* words admitted to this dictionary. Among these may be noticed *cockalorum, Delsartian,* delicatessen, electrocute, heliochromoscope, kodak (as a verb), linotype, and many others.

Then when we consider the question of the spelling of words, and the reliability of this dictionary in that regard, we find that all disputed spellings and pronunciations have been referred under the direction of Professor March to an Advisory Committee of fifty philologists in American, English, Canadian, Australian, and East Indian universities and representative professional writers and speakers of English. These differences of opinion concerning spelling are all recorded in a special section of the appendix, so that the reader can readily find out what is any man's opinion about a word.

The builders of this dictionary have shown a disposition to throw out the diphthongs. Thus they prefer to write Homeopathy, discarding the œ. The diphthong form is, however, given.

The spelling of geographical names is governed by the decisions of the United States Board of Geographical Names.

It is observed also that the final e of chemical words like bromine and quinine is omitted and our old friend sulphur is spelled, German fashion, Sulfur.

In giving the pronunciation of words, instead of the usual arbitrary way of expressing the character of the sounds, as seen in dictionaries in general, the builders of the Standard use the Scientific Alphabet prepared by the American Philological Association, in representing how words should be pronounced. This secures uniformity and removes the possibility of misunderstanding the actual sound intended to be demonstrated.

In giving definitions, the words of any particular department of knowledge are submitted to representatives of the science or art to which the word belongs, so as to insure accurate definitions. To quote the statement of the editors themselves: "The people to whom a term more especially belongs should have the right to say what they mean when they use that term." If, however, the term is used by others in a hostile sense, that meaning also is given. In this way the editors seek to get at the truth of every word.

Many more points of merit than are here given may be mentioned, but enough has been said to show the remarkable value of the book. This review taken in connection with that given of the first volume in April of last year and the numerous shorter notices that were published from month to month ought to carry such conviction to the minds of our readers, as would make them feel they could not go another day without a copy of this grand work.

As was said of it by the great astronomer, J. Norman Lockyer: "It passes the wit of man to suggest anything which ought to have been done that has not been done to make the dictionary a success."

